# Construction of Photothermal Intelligent Membranes for Point-of-Use Water Treatment

**DOI:** 10.3390/molecules29235733

**Published:** 2024-12-05

**Authors:** Hong Jiang, Jiarong Wang, Ying Liang, Chuan Qiao

**Affiliations:** MOE Key Laboratory of Deep Earth Science and Engineering, College of Architecture & Environmental Engineering, Sichuan University, Chengdu 610065, China; 2021223055155@stu.scu.edu.cn (H.J.); wjrscu@163.com (J.W.); liangying@scu.edu.cn (Y.L.)

**Keywords:** photothermal sterilization, point-of-use water treatment, microorganism rejection

## Abstract

For the removal of waterborne pathogens in remote areas and disaster emergency situations, point-source water treatment methods are more suitable. Photothermal sterilization is ideal for point-of-use (POU) systems, as it effectively eliminates pathogens without secondary pollution or bacterial resistance issues. By combining photothermal with membrane treatment, these membranes rapidly heat up under near-infrared (NIR) light, enabling both bacterial retention and sterilization. However, the decrease in membrane flux due to pore clogging during water treatment can significantly impact membrane efficiency. And adjusting the membrane pore size can significantly enhance flux recovery during cleaning, thereby restoring membrane efficiency. By synthesis multifunctional membranes that combine bacteria retention, sterilization, and flux recovery, it can meet the requirements of point-source water treatment: compact size, high efficiency, good safety, and easy maintenance. In this study, we developed an intelligent thermally responsive membrane (NIPAN@CNTs/PAN) by incorporating carbon nanotubes (CNTs) and forming a copolymer of N-isopropylacrylamide and polyacrylonitrile (NIPAN) coating into polyacrylonitrile membranes, offering dual functions of photothermal sterilization and self-cleaning. With 3% CNTs, the membrane achieves 100% sterilization within 6 min of NIR exposure, while the NIPAN layer’s added roughness boosts photothermal efficiency, achieving 100% sterilization within 4 min. Rinsing at 50 °C improved flux recovery from 50% to 87% and reduced irreversible fouling from 49.7% to 12.9%, demonstrating stable performance over multiple cycles and highlighting its potential for long-term use in practical POU applications.

## 1. Introduction

Waterborne pathogens pose a significant threat to public health, causing millions of deaths and countless illnesses worldwide each year [1]. Ensuring water security in remote areas where centralized water treatment is impractical, as well as in the aftermath of major natural disasters such as earthquakes, presents significant challenges. In sparsely populated and geographically dispersed regions, centralized water treatment systems are not only prohibitively expensive but also risk resource wastage due to low usage rates. Similarly, after large-scale natural disasters, damage to water treatment facilities and supply systems often leaves affected populations without access to safe water. In such scenarios, public supply systems and conventional water treatment facilities, such as water plants, are inadequate to meet these urgent needs. This highlights the necessity for point-of-use (POU) treatment systems, which are small-scale and decentralized solutions designed to provide immediate, high-quality water directly where it is needed [2,3,4,5]. POU treatment systems ensure access to safe drinking water at the point of use, effectively addressing the unique challenges posed by these circumstances. For POU water treatment, high efficiency, compact size, and avoidance of secondary pollution from chemical agents are essential [6]. Therefore, membrane treatment is an ideal method [7]. For POU applications, a multifunctional membrane that integrates bacteria retention, bacteria inactivation, and detachment of dead bacteria to prevent membrane fouling is required [8]. This is essential to meet the specific demands of POU: high safety, easy maintenance, and low fouling.

Bacteria retained during membrane filtration can form biofilms, clogging pores, reducing efficiency, and posing health risks [9]. While chemical disinfection methods and UV are common, they can be challenging and risky for POU applications due to secondary chemical pollution and health hazards for untrained personnel [10,11]. Recently, near-infrared-based photothermal sterilization has already been applied in the medical field [12,13,14]. It poses no radiation risk compared to UV, and does not produce secondary chemical pollution. With the decreasing costs of infrared lasers and photothermal agents, its application in water treatment has also become feasible [15,16,17]. However, photosensitizers are typically in particle form, so they need to be combined with membranes to form composite membranes that meet the safety requirements for POU applications.

Even if bacteria on the membrane are effectively killed, the bacterial fragments left behind can still clog the membrane pores, reducing filtration efficiency [18]. Thus, restoring membrane flux after bacterial removal requires not only killing the bacteria but also effectively cleaning the membrane by removing the attached bacterial fragments. Common cleaning methods, such as forward flushing, often fail due to these clogs, while backwashing requires skilled operators [19,20]. If the clogging issue during forward flushing can be resolved and membrane flux increased, the efficiency of forward flushing would improve. Flexibly adjusting pore size—smaller during filtration and larger during cleaning—could enhance the effectiveness of forward flushing [21]. In recent years, temperature-sensitive intelligent materials have been developed for various applications, with N-isopropylacrylamide (NIPAAm) being widely used due to its low critical solution temperature (LCST) and stability across different pH levels [22,23,24]. For example, Techawanitchai et al. grafted poly(NIPAAm-co-HMAAm) onto a magnetite/silica composite, creating an adjustable “switch” [25]. When exposed to an alternating magnetic field (AMF), the heat generated by magnetic particles induces a hydrophilic-to-hydrophobic phase change in the polymer, allowing for precise control of the elution profile. Therefore, by combining photothermal sterilization with the design of thermo-responsive membranes, we can not only achieve effective bacterial killing but also enhance membrane cleaning efficiency, thus improving reusability and meeting POU requirements for easy maintenance and minimal fouling.

In this paper, a photothermal sterilization and intelligent thermally responsive electrospun membrane was developed to effectively address the issues of bacterial growth on membranes and membrane contamination. A photothermally responsive electrospun nanofiber membrane was developed, incorporating carbon nanotubes (CNTs) and polyacrylonitrile (PAN) to enhance bacterial filtration and photothermal sterilization. Additionally, a temperature-sensitive copolymer of N-isopropylacrylamide and polyacrylonitrile (NIPAN) coating was electrosprayed onto the CNTs/PAN nanofiber membrane, utilizing NIPAAm and PAN grafts for thermal-responsive switching. Results show that the NIPAN@CNTs/PAN membrane effectively filters *E. coli*-LB and inhibits *E. coli*-LB growth through photothermal sterilization, preventing biofilm formation that can block membrane pores. The temperature-sensitive coating functions by reducing pore size at low temperatures to enhance filtration and increasing pore size during photothermal sterilization to improve flux, facilitating the forward flushing of trapped pollutants and enabling the membrane to be quickly ready for the next cycle of use.

## 2. Results and Discussion

### 2.1. Preparation and Characterization of Photothermal Nanofiber Membranes

**Figure 1:**
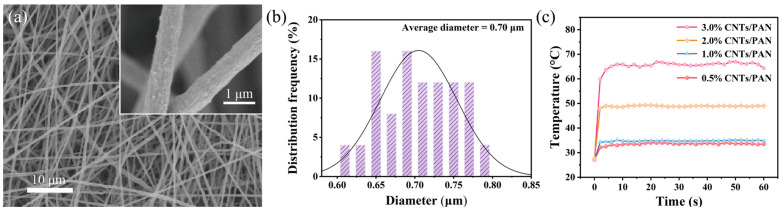
(**a**) SEM image of 3 wt% CNTs/PAN membranes. (**b**) Fiber diameter distribution of 3% CNTs/PAN membranes. (**c**) Optical heating capacity of CNTs/PAN membranes with different content of CNTs.

The material preparation followed these main principles: (1) strong microbial retention capacity, and (2) effective photothermal responsiveness and infrared sterilization capability. To meet these requirements, carbon nanotubes (CNTs) were chosen as the photothermal agent for doping into the fiber membrane material. CNTs are versatile nanomaterials with high elasticity, heat resistance, corrosion resistance, and strong light absorption, providing excellent photothermal effects [26]. Here, CNTs were doped into a PAN precursor spinning solution to produce uniformly blended CNTs-PAN membranes. After stirring and ultrasonic treatment, the CNTs were uniformly dispersed in the spinning solution. The increased viscosity of the solution, due to the addition of CNTs, helped prevent the gravitational settling of the CNTs, ensuring that the electrospinning process produced PAN membranes with a uniform distribution of CNTs. To examine the impact of varying CNT concentrations on fiber formation, different doping levels (0.5 wt%, 1 wt%, 2 wt%, and 3 wt%) were tested. Figure 1a and Figure S1 show SEM images of electrospun PAN membranes doped with various amounts of CNTs. The images reveal that the average fiber diameter increases with higher CNT doping levels. According to the statistical data in Figure 1b and Figure S2, average fiber diameters for 0.5 wt% to 3 wt% CNT content are 0.37 μm, 0.59 μm, 0.60 μm, and 0.70 μm, respectively, confirming a non-linear increasing trend of fiber diameter with CNT content. This is because, as the CNT content increases, the viscosity of the spinning solution gradually rises, causing stronger intermolecular entanglement. This force overcomes the electrostatic repulsion during the spinning process, reducing fiber stretching and resulting in larger fiber diameters [27]. Additionally, fiber surfaces become progressively rougher as doping levels rise, which is attributed to the viscosity of the spinning solution and the phase separation effect caused by solvent evaporation. To assess the effect of CNT doping on the material’s photothermal performance, blended membranes with different CNT concentrations were exposed to infrared light, and temperature changes over time were recorded with an infrared camera. As shown in Figure 1a, the photothermal capability of CNTs/PAN membranes improves as CNT content increases. Notably, with 3% CNT doping, the membrane surface temperature rose to 59.8 °C within only 2 s under 808 nm infrared irradiation, demonstrating excellent photothermal performance. Consequently, the 3% CNTs/PAN membrane was selected as the photothermal substrate for subsequent modifications and named the CNTs/PAN membrane.

### 2.2. Preparation and Characterization of Thermally Responsive Composite Membranes

**Figure 2:**
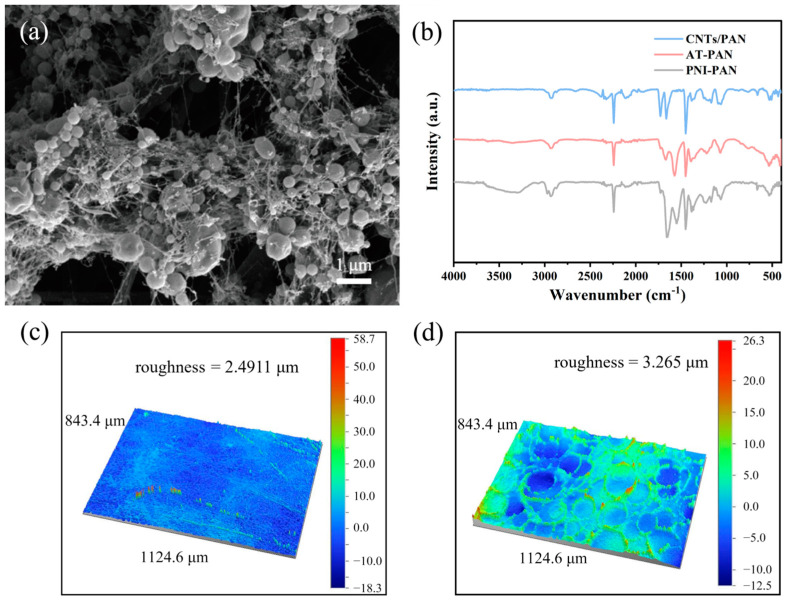
(**a**) SEM image of electrospray microspheres of 4% NIPAN@CNTs/PAN membranes. (**b**) FTIR spectra of 4% NIPAN@CNTs/PAN membranes. Three dimensional optical profilometer images of (**c**) CNTs/PAN and (**d**) 4% NIPAN@CNTs/PAN membranes.

By electrostatic spraying of NIPAN onto CNTs/PAN membranes, a temperature-sensitive coating was formed to enhance the anti-fouling properties of the membrane. To explore the effects of different NIPAN concentrations on membrane properties, CNTs/PAN substrate membranes were sprayed with electrospray fluids of 2%, 4%, and 6% concentrations, yielding 2% NIPAN@CNTs/PAN, 4% NIPAN@CNTs/PAN, and 6% NIPAN@CNTs/PAN membranes. Surface morphology analysis (Figure 2a and Figure S3) shows that all membranes feature spherical microspheres from the electrospray. The 2% NIPAN@CNTs/PAN membrane has fewer microspheres and contains numerous nano-segmented fiber-like structures dispersed or attached to them, likely due to the low conductivity at this concentration affecting spray efficiency. At 4% and 6%, finer nanowires appear between the microspheres with a higher microsphere count. Infrared analysis was used to verify material composition. In Figure 2b, AT-PAN (alkali-treated) and grafted NIPAN membranes show new peaks near 1550 cm^−1^ and 3450 cm^−1^, corresponding to broad hydroxyl peaks, N-H bonding groups, and C=C bonds. The 3450 cm^−1^ peaks are characteristic of NIPAAm and appear significantly enhanced, confirming successful NIPAN synthesis. Additionally, surface roughness analysis (Figure 2d) shows that the 4% NIPAN membrane material reaches a roughness of 3.265 μm, notably higher than that of the CNTs/PAN photothermal substrate membrane (2.49 μm in Figure 2c). According to Cassie’s model, the increased micro/nano structure due to electrospinning and electrospraying enhances the membrane’s surface roughness, promoting material hydrophilicity. According to Cassie/Wenzel model, the increased micro/nano structure due to electrospinning and electrospraying enhances the membrane’s surface roughness, promoting material hydrophilicity [28,29].

### 2.3. The Bacteria Filtration Capability of Thermally Responsive Composite Membranes

**Figure 3:**
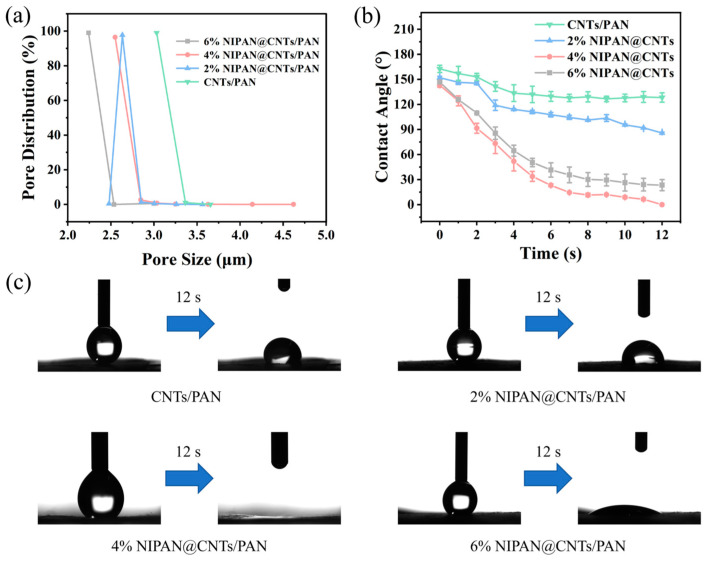
(**a**) Pore distribution and (**b**) time-varying water contact angles for CNTs/PAN and NIPAN@CNTs/PAN membranes with different NIPAN content. (**c**) Digital images for CNTs/PAN and NIPAN@CNTs/PAN membranes with different NIPAN content at initial and 12 s later.

The bacterial filtration capability of membrane materials is primarily determined by pore size, supported by hydrophilicity and surface electronegativity. During bacterial filtration of raw water, bacteria are captured and retained on the membrane through pore size sieving and charge attraction. Thus, pore size is a key factor in the bacterial retention capacity of the membrane system. The pore sizes of the CNTs/PAN membranes and fiber membranes containing NIPAN electrospray layers were measured using the bubble point method. As shown in Figure 3a, the pore sizes of the CNTs/PAN membrane were all greater than 3 microns, with 98.97% of the pores measuring around 3.031 μm. Adding NIPAN electrospray layers with varying precursor concentrations reduced the membrane’s pore size. More than 96% of the pores in the 2%, 4%, and 6% NIPAN@CNTs/PAN membranes were 2.633 μm (97.69%), 2.549 μm (96.41%), and 2.24 μm (98.99%), respectively. This occurs because the NIPAN electrospray forms microspheres on the fiber membrane surface, which fill the gaps within the fiber’s networked structure, decreasing the average pore size of the membrane system and enhancing bacterial retention. Although the NIPAN electrospray layer improves bacterial retention by reducing pore size, it may hinder filtration speed, leading to lower flux.

High flux is crucial for enhancing the efficiency of membrane materials in wastewater treatment applications involving microorganisms. Generally, increased pore size and improved hydrophilicity lead to higher operational flux in membrane materials. To assess this, the hydrophilicity of the materials was analyzed by measuring the contact angles of CNTs/PAN membranes with fiber membranes covered by electrospray layers at three different NIPAN concentrations over time. Due to the CNTs’ presence, CNTs/PAN membranes initially exhibited hydrophobicity [27,30], with an initial contact angle around 163° (Figure 3b). This angle briefly dropped within the first 4 s, then slowly decreased to 133.8° after 12 s, and remained at approximately 130° after 20 s. As seen in Figure 3b,c, all three composite fiber membranes with varying NIPAN concentrations exhibited significantly enhanced hydrophilicity compared to the original 3% CNTs/PAN at room temperature, i.e., below the lower critical solution temperature (LCST). The 4% NIPAN@CNTs/PAN composite fiber membrane demonstrated the highest hydrophilicity at 25 °C, with its water contact angle decreasing from 143° to 28° within just 5 s. In contrast, the initial CNTs/PAN fiber membrane showed a reduction in contact angle from 163° to 132° after the same period. The enhanced hydrophilicity from the temperature-sensitive NIPAN electrospray layer is likely due to the material’s compositional and morphological modifications. Alkali treatment introduces hydrophilic -OH groups to PAN, improving material hydrophilicity. Additionally, the increased roughness from the electrospray microsphere layer further enhances hydrophilicity, and the exposure of NIPAN’s hydrophilic end at room temperature contributes to this effect. This improved hydrophilicity supports greater contact between bacteria and the membrane, thus boosting the material’s retention capacity.

**Figure 4:**
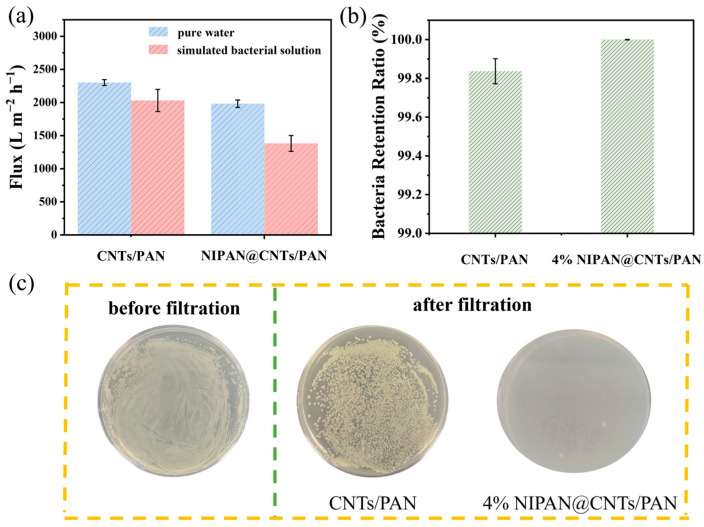
(**a**) Fluxes in water and simulated bacterial solutions and (**b**) retention ratio for both CNTs/PAN and 4% NIPAN@CNTs/PAN membranes. (**c**) Digital images expanded 20 h before and after filtration for both CNTs/PAN and 4% NIPAN@CNTs/PAN membranes.

The fluxes of the CNTs/PAN membranes and the fiber membranes with a 4% NIPAN electrospray layer (noted for their superior hydrophilicity) were further assessed in both pure water and a simulated bacterial solution, using *E. coli*-LB as the model bacterial contaminant. As shown in Figure 4a, fluxes were higher in pure water than in the simulated bacterial solution for both membrane types. This reduction in flux with the simulated bacterial solution is due to *E. coli*-LB accumulating on the membrane surface, which clogs pores, fosters membrane fouling, and subsequently lowers flux. Moreover, the 4% NIPAN@CNTs/PAN membranes experienced a decrease in flux for both pure water and simulated bacterial solutions when compared to CNTs/PAN membranes. With the addition of the NIPAN layer, the pore size decreased and surface roughness increased, leading to a slight reduction in water permeability. While this may slow down filtration slightly, it greatly benefits bacterial retention, as the smaller pore size enhances bacterial interception rates. Notably, the *E. coli*-LB filtration efficiency of the 4% NIPAN@CNTs/PAN membrane material increased substantially, with retention rates rising from 99.84% in CNTs/PAN membranes to nearly 100% (Figure 4b). Clearly, the reduction in membrane pore size and the increase in roughness favor bacterial retention, while the enhanced hydrophilicity of the membrane reduces bacterial adhesion and contamination.

### 2.4. Photothermal Sterilizability of Thermally Responsive Composite Membranes

**Figure 5:**
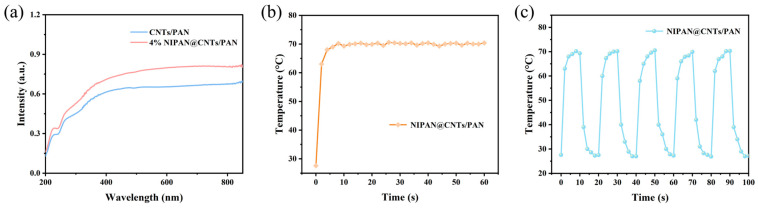
(**a**) UV-vis-NIR spectra of CNTs/PAN and 4% NIPAN@CNTs/PAN membranes. (**b**) Optical heating capacity of 4% NIPAN@CNTs/PAN membranes. (**c**) Cycling temperature curve of 4% NIPAN@CNTs/PAN membranes.

The photothermal capacity of the membrane material underpins its ability for photothermal sterilization. First, the UV-Vis-NIR absorption spectra of the 4% NIPAN@CNTs/PAN composite membrane were analyzed. As shown in Figure 5a, the composite fiber membrane coated with the temperature-sensitive NIPAN electrospray layer has a similar absorption spectrum trend to the original CNTs/PAN membrane. Both membranes exhibit minimal absorption in the UV range and substantial absorption in the visible and near-infrared ranges. However, the NIPAN-layered composite membrane demonstrates higher absorption in these bands compared to the original photothermal membrane without the electrospray layer. This enhanced light absorption is attributed to the introduction of the NIPAN electrospray microsphere layer, which contributes a multilevel rough structure to the membrane. Consequently, the surface roughness increased from 2.49 μm (Figure 2c) to 3.27 μm (Figure 2d), enhancing the material’s capacity for multi-stage reflection and absorption of incident light. The temperature rise of the membrane material under 808 nm light irradiation was measured, as shown in Figure 5b. The membrane heated to 63 °C in just 2 s under IR light, stabilizing around 70 °C after 8 s. This is an improvement compared to the CNTs/PAN base material and is directly linked to the increased light absorption due to the roughness added by the electrospray layer. Finally, temperature stability was evaluated by cycling infrared irradiation on and off. Results in Figure 5c show that in all five cycles, the membrane reached temperatures above 69.3 °C and cooled rapidly to room temperature within 10 s after the IR source was turned off. This rapid heating under infrared indicates the membrane’s efficient sterilization potential, while the quick cooling upon stopping the IR exposure ensures safety for long-term use. This capability is particularly beneficial for non-professional operators, such as those in remote areas, as it mitigates the risk of burns and promotes the safe, effective use of the membrane material.

**Figure 6:**
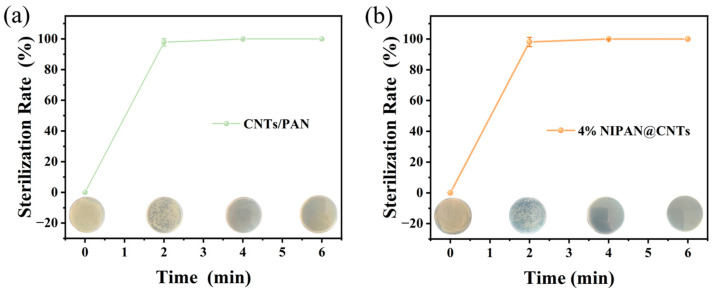
Sterilization rates upon NIR irradiation at different time for both (**a**) CNTs/PAN and (**b**) 4% NIPAN@CNTs/PAN membranes.

With the material’s strong photothermal capabilities confirmed, further testing was necessary to verify its bactericidal effectiveness under infrared irradiation. *E. coli* in an *E. coli*-LB solution was used to simulate bacterial entrapment on the membrane, with IR sterilization experiments conducted on both CNTs/PAN and 4% NIPAN@CNTs/PAN membranes. As shown in Figure 6a,b, infrared irradiation was applied for various durations, and the bactericidal efficiency of each membrane was assessed through a plate-coating method. For the CNTs/PAN membranes, a positive correlation between sterilization rate and irradiation time was observed; the photothermal sterilization rate steadily increased with prolonged exposure. Thanks to the efficient photothermal effect of CNTs, the CNTs/PAN membrane achieved a 97.8% sterilization rate after just 2 min, reaching nearly 100% after 6 min. In contrast, the 4% NIPAN@CNTs/PAN membrane exhibited an even higher initial bactericidal rate of 98.1% after only 2 min of irradiation. This enhanced sterilization is due to the introduction of the NIPAN electrospray layer, which increases surface roughness, thereby enhancing light absorption and achieving higher surface temperatures under the same infrared exposure. This increased temperature accelerates the sterilization process, allowing for superior bactericidal efficiency. Such sustained and intensified sterilization capacity enables the material to effectively eliminate entrapped bacteria, preventing their growth and subsequent biofouling, which would otherwise lead to significant membrane contamination.

### 2.5. Contamination Resistance of Thermally Responsive Composite Membranes

**Figure 7:**
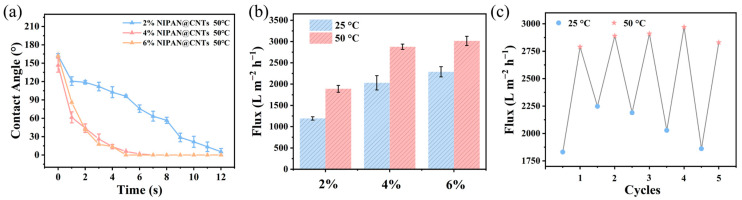
(**a**) Time-dependent static water contact angles in air of NIPAN@CNTs/PAN membranes with different NIPAN content above LCST (50 °C). (**b**) Water fluxes of NIPAN@CNTs/PAN membranes with different NIPAN content below (25 °C) and above LCST (50 °C). (**c**) Cycling pure water flux of 4% NIPAN@CNTs/PAN below (25 °C) and above LCST (50 °C).

To explore the impact of the thermosensitive substance NIPAAm on the membrane’s flux, it was essential to analyze changes in the water contact angle over time both above and below the LCST for the three composite fiber membranes with different NIPAN electrospray concentrations. The contact angle changes above the LCST were subsequently measured for each material. As shown in Figure 7a, all three NIPAN@CNTs/PAN membranes displayed enhanced hydrophilicity at 50 °C compared to 25 °C (Figure 3b). For instance, with the 4% NIPAN@CNTs/PAN membrane, the contact angle rapidly decreased from an initial 143° to 91° within 2 s at 25 °C. At 50 °C, however, the contact angle dropped even more drastically, from 146° to 44° within 2 s, indicating superior hydrophilicity. Although temperatures above the LCST, such as 50 °C, cause NIPAN chains to collapse and expose their hydrophobic ends, theoretically reducing liquid wettability, elevated temperatures also increase the kinetic energy of water molecules, thereby raising intermolecular forces and decreasing surface tension—factors that enhance wettability and lower the contact angle. Additionally, the NIPAN collapse at higher temperatures may contribute to slight increases in surface roughness, further promoting hydrophilicity. This indicates that the combined hydrophilic effects—arising from chemical interactions, kinetic forces, and morphological changes of NIPAN at elevated temperatures—outweigh the hydrophobic effect introduced by the exposure of hydrophobic ends.

The water flux increased with higher NIPAN content in the electrospray precursor solution, likely due to NIPAN’s pore-forming effect, which changes pore structure (Figure 7b). For typical CNTs/PAN membranes, flux slightly increases with temperature, primarily because water viscosity decreases at higher temperatures. However, for composite fiber membranes containing a thermoresponsive electrospray layer, the flux increase is much more pronounced. Specifically, the 2% NIPAN@CNTs/PAN membrane showed a 58% flux increase at 50 °C compared to 25 °C, which far exceeds the effect of temperature alone. This substantial flux increase is attributed to changes in membrane permeability due to NIPAN’s temperature response, which results in pore opening above the LCST as NIPAAm chains contract. Below the LCST, extended NIPAAm chains cover some membrane pores, reducing both pore size and flux. Among the composite membranes, the 4% NIPAN fiber membrane excelled in hydrophilicity and enhanced water flux post-heating, and also showed cost-effectiveness, making it ideal for further flux testing under cyclic temperature changes. As seen in Figure 7c, over five cycles, the 4% NIPAN@CNTs/PAN membrane displayed consistent water flux at both above and below the LCST, with stable values of around 2250 L m^−2^ h^−1^ at 25 °C and over 2750 L m^−2^ h^−1^ at 50 °C. This remarkable stability and reversibility suggest broad applications in fields like intelligent separation, filtration, and sterilization. For example, at low temperatures, the membrane’s reduced pore size enables effective bacterial retention, while at higher temperatures, the enlarged pores facilitate contaminant flushing from the clogged membrane, restoring flux.

Smaller pore sizes lead to higher osmotic pressure and thus the flux decreases during gravity-driven filtration. However, smaller pores also enhance bacterial retention. During prolonged use, bacteria trapped on the surface and within the pores of membrane materials can accumulate, proliferate, and form localized biofilms. This biofilm formation can significantly clog the pores, reducing flux and filtration efficiency—posing a challenge for the long-term usability of membrane materials. Therefore, effective contaminant removal from clogged pores to restore flux is essential to the membrane’s durability. The introduction of temperature-sensitive NIPAN provides a solution to this issue. At lower temperatures (<LCST), the composite fiber membrane has smaller pore sizes conducive to contaminant separation. Above the LCST, the contracted NIPAN chains expand the membrane pores, facilitating the rapid rinsing of retained contaminants. This effect is largely influenced by the properties of NIPAN chains and the behavior of heated water molecules [31]. Building on the above findings, NIPAN@CNTs/PAN composite fiber membranes exhibit intelligent, temperature-sensitive properties. When the membrane temperature rises from 25 °C to 50 °C, its hydrophilicity improves, and the pore size increase. These changes boost water flux and accelerate the rinsing process. Furthermore, as the temperature rises, the kinetic energy of water molecules increases, allowing them to penetrate the membrane pores more effectively and carry away contaminants [32]. To confirm NIPAN’s role in flux recovery through intelligent cleaning, this property was tested for microbial retention and flux restoration using a simulated bacterial solution. The study involved the *E. coli*-LB retention under gravity conditions and cleaning of the contaminated membrane with fresh water at 25 °C and 50 °C. During the *E. coli*-LB filtration process, the membrane flux gradually declined as contaminants accumulated. After reaching a certain level of fouling, the membrane was rinsed with water at 25 °C and 50 °C. Notably, this rinsing process differs from the conventional “backwash” method used in water treatment plants. Instead, a “forward rinse” was employed, where water flows in the same direction as the filtration process.

**Figure 8:**
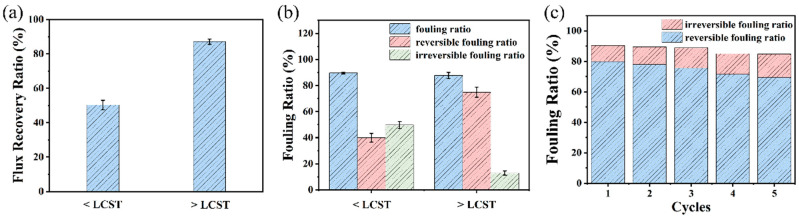
(**a**) Fluxes recovery ratio (FRR) of 4% NIPAN@CNTs/PAN membranes below LCST and above LCST. (**b**) Fouling ratio (Rt), reversible fouling ratio (Rr) and irreversible fouling ratio (Rir) of 4% NIPAN@CNTs/PAN membranes below LCST and above LCST. (**c**) Cycling antifouling tests of the 4% NIPAN@CNTs/PAN membrane.

Flux recovery ratios, total fouling ratios, irreversible fouling ratios, and reversible fouling ratios were calculated to compare recovery performance under these conditions. As shown in Figure 8a, rinsing with 50 °C water restored 87% of the membrane flux, compared to only 50% with 25 °C water. Higher flux recovery reflects the ability to retain filtration capacity post-rinsing, improving the material’s reusability. The total fouling, reversible fouling, and irreversible fouling ratios were also analyzed under the two cleaning temperatures. After contamination and rinsing, some flux was recovered (reversible fouling), while some contamination remained (irreversible fouling), indicating severe, long-term clogging that restricts the membrane’s processing capacity. In practical applications, it is ideal for membrane fouling to be reversible, as this allows contaminant removal and flux restoration, supporting long-term functionality. Figure 8b shows that while total fouling ratios were similar across rinsing temperatures, the reversible fouling ratio was significantly higher for 50 °C rinsing (75%) than for 25 °C rinsing (39%), indicating that most of the contaminants clogging the pores were removed with hot water. To evaluate the membrane’s long-term performance, the 4% NIPAN@CNTs/PAN composite fiber membrane underwent cyclic fouling-flushing tests. As seen in Figure 8c, stable reversible fouling and total fouling ratios were maintained across five cycles, demonstrating the feasibility and stability of using hot water rinsing to restore membrane flux, which is beneficial for long-term operation. This hot-water flushing approach is broadly applicable: temperature-induced changes in pore size can remove various retained contaminants, including microorganisms. For instance, Wei et al. used a temperature-sensitive PNIPAM-coated fiber membrane to remove oil droplets, restoring the membrane’s flux [33]. As water temperature increased from 20 °C to 50 °C, flux recovery improved from 59.3% to 82.8%, and irreversible fouling dropped from 40.7% to 17.2%. This technique has promising potential for use in practical water treatment systems for a range of contaminant removal needs.

It is worth noting that while the current performance of NIPAN@CNTs/PAN membranes effectively addresses bacterial retention and eradication in POU water treatment, the planar membrane morphology limits its filtration efficiency, as this is directly tied to the membrane area. Therefore, there is room for improvement in filtration performance. Drawing inspiration from membrane treatment processes used in water plants, the filtration efficiency could be significantly enhanced by altering the membrane’s morphology through structural design, such as transforming planar membranes into hollow fiber or tubular forms. Additionally, considering the membrane’s photothermal sterilization capabilities, a comb- or brush-like arrangement of hollow tubular membranes could be employed. This design would maximize exposure to near-infrared irradiation during sterilization, ensuring effective coverage across all membrane surfaces and optimizing the sterilization effect.

## 3. Materials and Methods

### 3.1. Synthesis

#### 3.1.1. Synthesis of CNTs/PAN Substrate Membranes

Weigh a measured amount of dry CNT powder (Aladdin, Shanghai, China) (1%, 2%, 3%, *w*/*v*) into a 25 mL threaded triangular flask, add N,N-Dimethylformamide (DMF) (Chron Chemicals, Chengdu, China) and sonicate at 35 °C, 40 kHz, and 90 W for 2 h to obtain a uniformly dispersed CNT solution. Add dry PAN powder (Aladdin, Shanghai, China) (12%, *w*/*v*) to the CNT solution, stirring at 700 rpm in a 25 °C water bath for 12 h. Transfer the solution to a sample bottle to allow defoaming. Load the electrospinning solution into two identical 5 mL medical syringes equipped with 21-G flat-tip stainless steel needles. Electrospinning was performed for 5 h under conditions of 10 kV, a temperature of 35 ± 5 °C, humidity of 45 ± 5%, a feed rate of 0.04 mm/min, and a 17 cm needle-to-drum distance. The CNTs/PAN nanofiber membrane on silicone oil paper was then removed and dried in a 60 °C oven for 6 h.

#### 3.1.2. Synthesis of Thermally Responsive Composite Membranes

Dissolve 10 g of 2,2-Azobisisobutyronitrile (AIBN) (Aladdin, Shanghai, China) in 100 mL of ethanol (Chron Chemicals, Chengdu, China) preheated to 50 °C in a conical flask and stir until fully dissolved. Filter the solution hot to remove insoluble impurities. Let the filtrate cool to room temperature, then refrigerate at 4 °C to recrystallize. Once crystallized, filter, dry the recrystallized product in a vacuum oven at 25 °C for 24 h, and store it in a light-protected refrigerator.

Dissolve 4 g of NIPAAm monomer (Aladdin, Shanghai, China) in a 1:1 (*v*/*v*) acetone (Chron Chemicals, Chengdu, China) and n-hexane (Chron Chemicals, Chengdu, China) solution at 45 °C with stirring, then filter hot to remove impurities. Cool the filtrate to room temperature, refrigerate at 4 °C for 6 h, then filter to obtain a white powder or solid. Dry this in a vacuum oven at 40 °C for 2 h, grind, then bake for an additional 6 h in the vacuum oven. Store the resulting powder in an airtight vial in the refrigerator.

Dissolve 4.9 g of KOH (Chron Chemicals, Chengdu, China) in 34 mL ultrapure water, add 1 mL ethanol, and mix thoroughly at room temperature. Add 3.5 g of pre-dried PAN powder and stir at 60 °C for 30 min. Filter the solid, dry it at 50 °C for 6 h in a vacuum oven, and grind to obtain alkali-treated AT-PAN for storage.

Dissolve AT-PAN in DMF (1:12, *w*/*v*), add purified NIPAAm (1:12, *w*/*v*) and AIBN (0.4 wt% relative to the reaction monomer), vent nitrogen for 30 min, then heat at 70 °C under nitrogen for 12 h. Add methanol (2× volume) and stir gently to precipitate the NIPAAm and AT-PAN copolymer. Filter, wash with deionized water, and dry the copolymer at 50 °C in a vacuum oven for 4 h. Grind and dry further for 8 h to yield NIPAAm-PAN copolymer (NIPAN).

Place varying masses of NIPAN (2%, 4%, 6%, *w*/*v*) in a threaded flask, add DMF, and stir at 50 °C at 700 rpm until uniform. Load this electrospray precursor solution into a 5 mL syringe with a 21-G needle for electrospinning. Place the CNTs/PAN membrane prepared from Section 3.1.1 at the receiving end and electrospray under 10 kV, 0.04 mm/min speed, 45 ± 5% humidity, and 25 ± 5 °C for 4 h. After 4 h, carefully remove the electrosprayed fiber membrane and dry it at 60 °C for 6 h.

### 3.2. Characterization

The microstructure of the electrospun fiber membranes was examined with a JSM-7500F Field Emission Scanning Electron Microscope from JEOL, Tokyo, Japan, using an accelerating voltage of 5–15 kV and a sample gold-coating time of approximately 45 s. FTIR spectra were obtained using a Spectrum Two FTIR spectrometer from PerkinElmer, Waltham, MA, USA.

Water contact angle (WCA) was measured as a parameter of wettability using a JC2000D4B contact angle meter from Shanghai Zhongchen Digital Technology Equipment Co. (Shanghai, China). The fiber membrane was fixed onto a slide with double-sided tape to ensure surface flatness before testing. For experiments above LCST (50 °C), an electric heating pad was applied to test the WCA at different temperatures.

Pore size distribution was determined using the bubble point method. Membrane discs, 25 mm in diameter, were immersed in a profiling liquid for 5 min to ensure full pore saturation. Measurements were then performed with a Porolux 1000 capillary flow pore size analyzer from Porometer, Odelzhausen, Germany.

### 3.3. Flux Measurement

Membranes were cut into 20 mm diameter discs and placed atop a sand core filter, secured between a glass tube and an Erlenmeyer flask with a clamp. Pre-wetted with deionized water, the membranes operated under gravity-driven flow. The flux value (L m^−2^ h^−1^) was calculated using:

(1) Flux=V/At

where V is the filtered solution volume (L), A is the filtration area (m^2^), and t is the filtration time (h).

### 3.4. Bacterial Filtration Test

*Escherichia coli* (CICC10899), a common pathogenic bacterium, served as the target microorganism. A 20 mL diluted *E. coli* suspension (~10^6^ CFU/mL) was filtered using a custom laboratory device, with gravity as the sole driving force. Following filtration, 100 μL of the filtrate was plated on LB agar and incubated at 37 °C for 20 h. Colony images were taken for bacterial count analysis.

### 3.5. Photothermal Sterilization Rate Test

An 808 nm infrared laser (MDL-H-808-5W, Changchun New Industry Optoelectronics Technology Co., Ltd., Changchun, China) at 0.95 W was used, with an end collimator aperture of 6–8 mm and a divergence angle below 19 mrad. The membrane was positioned 53 cm beneath the laser to measure photothermal response, with temperature changes recorded over time using a PTi120 infrared camera from Fluke, Everett, WA, USA.

To assess bactericidal efficiency, *E. coli* (CICC 10899) was cultured in LB at 37 °C for 12 h and diluted to ~10^6^ CFU/mL. A sterilized 6 mm diameter membrane was inoculated with 10 μL of bacterial solution, then irradiated at 808 nm (1.2 W, 53 cm distance) for varied durations. Following irradiation, the membrane was transferred to PBS buffer in a centrifuge tube, sonicated for 3 min (30 ± 5 °C, 80 W), and then 100 μL of the sonicated bacterial suspension was plated on LB agar and incubated at 37 °C for 20 h to evaluate sterilization performance.

### 3.6. Anti-Fouling Test of Thermally Responsive Composite Membranes

The membrane’s initial flux *P*_0_ (L m^−2^ h^−1^) was measured by filtering deionized water under 0.15 bar pressure for 5 min, then continuing for 30 min by gravity alone. Subsequently, a bacterial solution (~10^6^ CFU) was filtered for 60 min to obtain flux *P*_1_ (L m^−2^ h^−1^). The membrane was cleaned with deionized water at varying temperatures (25 °C, 50 °C) for 10 min at 0.15 bar, followed by a 30 min measurement of the recovered pure water flux *P*_2_ (L m^−2^ h^−1^). Cleaning cycles were repeated five times to calculate the flux recovery rate (FRR) as follows:

(2) $$\mathrm{FRR}=\frac{P_{2}}{P_{0}}\times 100\%$$

To evaluate the recovery capability of thermally responsive membranes, fouling metrics such as total fouling ratio (Rt), reversible fouling ratio (Rr), and irreversible fouling ratio (Rir) were calculated as follows:

(3) $$\mathrm{Rt}=(1-\frac{P_{1}}{P_{0}})\times 100\%$$

(4) $$\mathrm{Rr}=(\frac{P_{2}-P_{1}}{P_{0}})\times 100\%$$

(5) $$\mathrm{Rir}=(\frac{P_{0}-P_{2}}{P_{0}})\times 100\%$$

## 4. Conclusions

In summary, to efficiently clear contaminants from membrane pores and restore flux after fouling, the thermo-responsive composite membrane NIPAN@CNTs/PAN was developed using electrospinning and electrostatic spraying, incorporating photothermal CNTs and temperature-sensitive NIPAN. The robust photothermal effect of CNTs enables effective infrared sterilization, quickly eliminating trapped bacteria to prevent clogging and ensure membrane efficiency. The temperature sensitivity of NIPAN results in a dual function: at low temperatures, the membrane retains bacteria with a smaller pore size, while at high temperatures, it allows larger pores and higher flux for effective flushing, and flux recovery rose from 50% to 87%, while irreversible fouling dropped from 49.7% to 12.9%. Most contaminants were successfully removed at elevated temperatures, allowing stable flux recovery and reversible fouling in cyclic tests, confirming the material’s excellent reusability. Therefore, the NIPAN@CNTs/PAN membrane has functions such as bacterial retention, photothermal sterilization, and thermosensitive regulation of pore size to flush away bacteria. It is suitable for POU applications that require safety, convenience in maintenance, and minimal pollution.

## Data Availability

The data supporting the findings of this study are available upon request from the corresponding author.

## Supplementary Materials

The following supporting information can be downloaded at: https://www.mdpi.com/article/10.3390/molecules29235733/s1, Figure S1: SEM image of 0.5 wt%, 1 wt% and 2 wt% CNTs/PAN membranes; Figure S2: Fiber diameter distribution of 0.5 wt%, 1 wt% and 2 wt% CNTs/PAN membranes; Figure S3: SEM image of electrospray microspheres of 2% and 6% NIPAN@CNTs/PAN membranes; Figure S4: Diameter distribution diagrams of electrospray microspheres of 2%, 4% and 6% NIPAN@CNTs/PAN membranes.
