# Supplementary material for: Construction of Photothermal Intelligent Membranes for Point-of-Use Water Treatment
Source: Molecules. 2024 Dec 5;29(23):5733. doi: 10.3390/molecules29235733 (PMC11643236; doi:10.3390/molecules29235733)
Supplement: Supplementary file 1 [file molecules-29-05733-s001.zip › molecules-3328246-supplementary.pdf]

Supplementary Material

Construction of Photothermal Intelligent Membranes for Point-of-Use Water Treatment

Hong Jiang, Jiarong Wang, Ying Liang and Chuan Qiao *

MOE Key Laboratory of Deep Earth Science and Engineering, College of Architecture & Environmental Engineering, Sichuan University, Chengdu 610065, China; 2021223055155@stu.scu.edu.cn (H.J.); wjrscu@163.com (J.W.); liangying@scu.edu.cn (Y.L.)

* Correspondence: chuan.qiao@scu.edu.cn

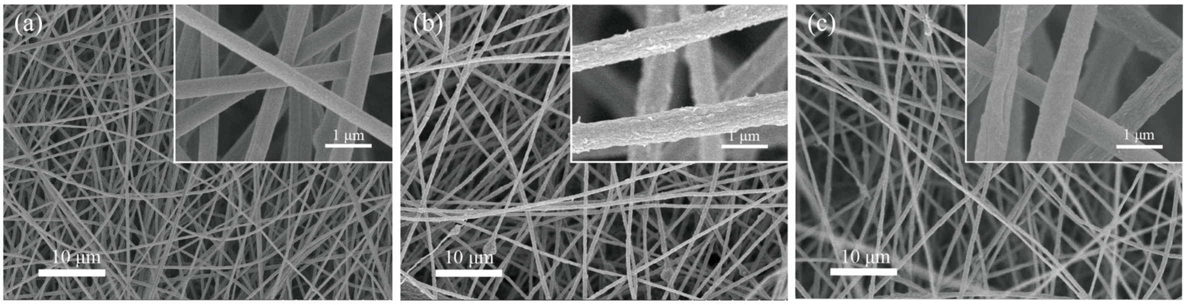

Figure S1. SEM image of (a) 0.5 wt%, (b) 1 wt% and (c) 2 wt% CNTs/PAN membranes.

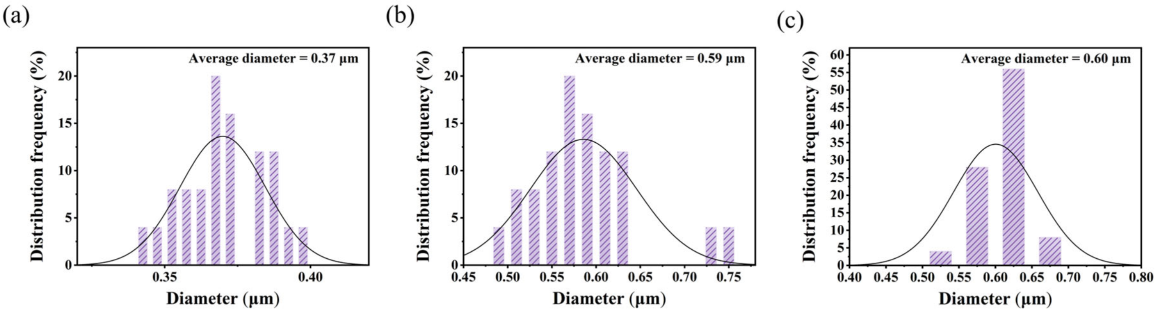

Figure S2. Fiber diameter distribution of (a) 0.5 wt%, (b) 1 wt% and (c) 2 wt% CNTs/PAN membranes.

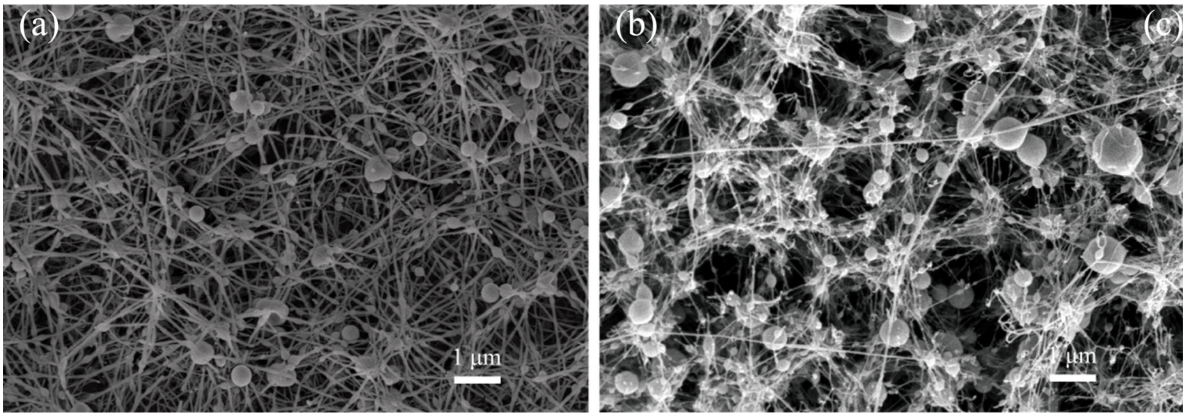

Figure S3. SEM image of electrospray microspheres of (a) 2% and (b) 6% NIPAN@CNTs/PAN membranes.

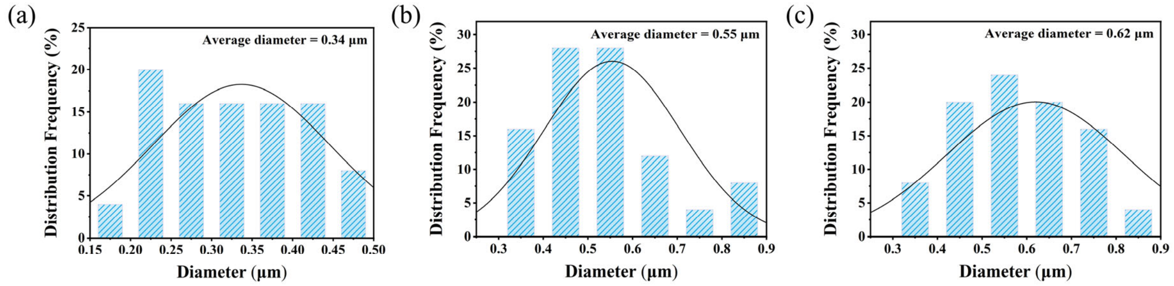

Figure S4. Diameter distribution diagrams of electrospray microspheres of (a) 2%, (b) 4% and (c) 6% NIPAN@CNTs/PAN membranes.
